# Phytochemical-Based Nanoantioxidants Stabilized with Polyvinylpyrrolidone for Enhanced Antibacterial, Antioxidant, and Anti-Inflammatory Activities

**DOI:** 10.3390/antiox13091056

**Published:** 2024-08-30

**Authors:** Hyeryeon Oh, Jin Sil Lee, Hyojung Park, Panmo Son, Byoung Seung Jeon, Sang Soo Lee, Daekyung Sung, Jong-Min Lim, Won Il Choi

**Affiliations:** 1 Bio-Convergence Materials R&D Division, Korea Institute of Ceramic Engineering and Technology, 202 Osongsaengmyeong 1-ro, Heungdeok-gu, Cheongju 28160, Republic of Korea; hyeryeon.oh@kicet.re.kr (H.O.); jslee92@kicet.re.kr (J.S.L.); hyojungp818@gmail.com (H.P.); spm1006@kicet.re.kr (P.S.); a1trust@kicet.re.kr (B.S.J.); dksung@kicet.re.kr (D.S.); 2 School of Materials Science and Engineering, Gwangju Institute of Science and Technology, 123 Cheomdangwagi-ro, Buk-gu, Gwangju 61005, Republic of Korea; 3 Department of Applied Bioengineering, Graduate School of Convergence Science and Technology, Seoul National University, 1 Gwanak-ro, Gwanak-gu, Seoul 08826, Republic of Korea; 4 Department of Electronic Materials, Devices, and Equipment Engineering, Soonchunhyang University, 22 Soonchunhyang-ro, Shinchang-myeon, Asan 31538, Republic of Korea; gnpzl@naver.com; 5 Department of Chemical Engineering, Soonchunhyang University, 22 Soonchunhyang-ro, Shinchang-myeon, Asan 31538, Republic of Korea

**Keywords:** phytoncide, essential oil, encapsulation, micelle, antioxidant, anti-inflammation

## Abstract

Despite the inhibitory effect of phytoncide (Pht) on food-borne pathogenic bacterial growth, the hydrophobic nature and susceptibility to biodegradation under physiological conditions limits its applications. Here, we developed Pht-loaded polyvinylpyrrolidone (PVP) micelles (Pht@PVP MC) via micelle packing. Pht was solubilized using different types of PVP as micellar vehicles. The as-prepared Pht@PVP MCs were characterized using dynamic light scattering and transmission electron microscopy. The sizes of the Pht@PVP MCs were controlled from 301 ± 51 to 80 ± 3 nm by adjusting the PVP content. The polydispersity index of Pht@PVP MC was between 0.21 ± 0.03 and 0.16 ± 0.04, indicating homogeneous size. A colony-counting method was employed to evaluate the improvement in antibacterial activity after Pht encapsulation in PVP micelles. The reactive oxygen species (ROS)-scavenging activity and anti-inflammatory efficacy of Pht@PVP MC were analyzed in a concentration range of 10–100 μg/mL by evaluating in vitro ROS and nitric oxide levels using DCFDA and Griess reagents. PVP with both hydrophobic and hydrophilic moieties improved the aqueous solubility of Pht and stabilized it via steric hindrance. Higher-molecular-weight PVP at higher concentrations resulted in a smaller hydrodynamic diameter of Pht@PVP MC with uniform size distribution. The spherical Pht@PVP MC maintained its size and polydispersity index in a biological buffer for 2 weeks. Pht@PVP MC exhibited enhanced antibacterial activity compared to bare Pht. The growth of *Staphylococcus aureus* was effectively inhibited by Pht@PVP MC treatment. Furthermore, biocompatible Pht@PVP MC exhibited dose-dependent antioxidant and anti-inflammatory activities in vitro. Overall, Pht@PVP MC is an effective alternative to synthetic antibacterial, antioxidant, and anti-inflammatory chemicals.

## 1. Introduction

Phytoncide (Pht) is a complex mixture of essential oils (EOs) containing naturally derived volatile compounds used by plants in their antioxidant defense systems against insects and microorganisms [1]. Pht comprises more than 300 different chemical classes of compounds, including alcohols, ethers, aldehydes, ketones, esters, amines, amides, phenols, heterocycles, and terpenes [2]. They synergistically contribute to the distinctive scents and biological activities of oils. In particular, terpenes and terpenoids are major components, having antibacterial and antioxidant activity [3]. Pht exerts antibacterial activity by disrupting cell membrane integrity, inhibiting enzymatic activity, and interfering with nucleic acid synthesis. Lipophilic Pht facilitates the penetration of the bacterial cell membrane. Thus, exposure to Pht causes the death of the pathogenic bacteria as a result of binding with bacterial proteins and cell lysis [4]. It also inhibits enzymes involved in inflammation and neutralizes free radicals to reduce oxidative stress. It enables the transfer of electrons or hydrogen atoms to free radicals that can cause cellular damage [5]. Further, Kim et al. summarized their anti-inflammatory effects via signal transduction; autophagy; and regulation of pro-inflammatory mediators, transcription factors, and oxidative stress [6]. Pht possesses several therapeutic properties, including immunomodulatory, anticancer, antioxidant, anti-inflammatory, and antimicrobial properties [7,8]. Therefore, Pht has been explored as an alternative to synthetic medicines, demonstrating its efficacy without adverse effects. It exhibits anti-asthmatic activity [9], accelerates wound regeneration [10], and inhibits dysregulated reactive oxygen species (ROS) secretion [11]. In food processing and preservation, Pht can reportedly prolong the shelf life of food by suppressing oxidation and pathogenic bacterial growth [12].

However, Pht is highly hydrophobic, resulting in poor bioavailability in aqueous solutions and susceptibility to chemical degradation when exposed to harsh conditions [13,14]. Therefore, strategies are required to enhance the stability, hydrophilicity, and efficacy of Pht. Encapsulation of EOs in nanoparticles has been shown to protect EOs from the external environment, preserve their bioavailability, and prevent oxidation while facilitating controlled delivery [15]. It reduces their volatility and ensures longer periods of biological activity. While the delivery system minimizes the direct contact of Pht with biological tissues to reduce the risk of toxicity, increased solubility and enhanced absorption of Pht may improve its biological efficacy. Lipid-based nanocarriers, inorganic nanoparticles, and core-shell nanocapsules have been used for encapsulation. Consequently, polymeric nanoparticles have been extensively studied as vehicles for EOs, showing excellent biocompatibility and satisfactory bioactivity under physiological conditions [16,17]. Usage of chitosan, poly(lactic-co-glycolic) acid, or poly(ε-caprolactone) nanoparticles as a delivery system could control the physicochemical characteristics and the release rate of EOs, leading to enhanced biological activity [18,19,20]. Successful encapsulation of EOs may support their effective use in a variety of applications.

Among the various polymers, polyvinylpyrrolidone (PVP) is a promising candidate for enhancing the bioavailability of Pht through micelle packing. Since its approval by the United States Food and Drug Administration, PVP has been extensively used in various technological and pharmaceutical applications [21]. Its general safety and high biocompatibility offer superior advantages as a delivery system. Further, it is effective in increasing the solubility of hydrophobic active ingredients, making them more effective in biological conditions. Owing to its amphiphilic nature, it can encapsulate hydrophobic EOs inside the core of a micelle, providing a shielding effect. Its structure, featuring polar amide groups along with apolar methylene and methine groups, renders PVP soluble in both aqueous and nonaqueous solutions. PVP, through robust hydrogen bonds, facilitates the aqueous dispersibility of hydrophobic natural substances [22]. PVP complexes with active materials and improves their solubility and stability by preventing crystallization and ensuring uniform distribution in biological environments. Thus, encapsulating Pht with PVP polymers may enhance its bioefficacy under physiological conditions; to the best of our knowledge, no such study has been conducted.

In this study, Pht oil from *Chamaecyparis obtusa* was encapsulated in PVP micelles (Pht@PVP MC) to improve its aqueous solubility and bioavailability for applications in nutraceuticals, biocides, cosmeceuticals, and therapeutic nanomaterials. PVP of different molecular weights (MWs) was employed at various concentrations for the micellar solubilization of Pht. Hydrodynamic diameters, polydispersity indices, and zeta potentials of the resulting Pht@PVP MC were analyzed using dynamic light scattering (DLS). Transmission electron microscopy (TEM) was used to observe the micelle morphology. Improvement in antibacterial activity due to PVP encapsulation was assessed using the colony-counting method. In vitro assays were performed to analyze the ROS-scavenging activity and anti-inflammatory efficacy of Pht@PVP MC.

## 2. Materials and Methods

### 2.1. Materials

Pht from *C. obtusa*, PVP polymers of different chain lengths (MW = 10, 40, 360, and 1300 kDa), and lipopolysaccharide (LPS) from *Escherichia coli* O111:B4 were purchased from Sigma-Aldrich (St. Louis, MO, USA). Hydrogen peroxide (H_2_O_2_, 30%), 2′,7′-dichlorodihydrofluorescein diacetate (H_2_DCFDA), and Griess reagent were purchased from Junsei Chemical Co. (Tokyo, Japan), Invitrogen (Carlsbad, CA, USA), and Abcam (Cambridge, UK), respectively. Deionized (DI) water and phosphate-buffered saline (PBS) were obtained from HyClone (Logan, UT, USA). The gram-positive bacterium *Staphylococcus aureus* ATCC 6538 was purchased from the American Type Culture Collection (Manassas, VA, USA).

### 2.2. Preparation and Characterization of Pht@PVP MC

Chemical characterization of Pht was performed using a gas chromatography device coupled with a mass spectrometry detector (GC-ToFMS; PEGASUS BT 4D GCXGC-ToFMS, Leco, St. Joseph, MI, USA) equipped with a capillary column (Rxi-5MS, 30 m × 0.25 mm × 0.25 μm film), an injector at 250 °C, and a helium gas flow rate of 1.0 mL/min. The initial temperature of the column furnace was set to 40 °C for 2 min, after which it was heated to 280 °C at a rate of 20 °C/min and maintained at 280 °C for 6.5 min. The total run time was 20 min. The Pht samples were diluted in hexane and analyzed using GC-ToFMS. The data processing and display of GC-ToFMS chromatograms were performed using the integrated LECO ChromaToF software (v.5.55.29.0). Deconvoluted signal peak apexes were automatically detected. Each detected peak was annotated by matching its mass spectrum to the reference spectra in the database. For peak identification in this study, the NIST mainlib and replib EI libraries were utilized. The mass similarity threshold for peak annotation was set at 800. After the identification of the majority of the Pht compounds, PVP of different chain lengths and concentrations was used to encapsulate the hydrophobic Pht. Briefly, Pht (20 mg) and PVP (40, 80, and 120 mg) were dissolved in DI water at EO/polymer weight ratios of 1:2, 1:4, and 1:6. The solutions were allowed to react for 12 h with rotatory shaking at 4 °C. Excess unreacted residues were removed via spin filtration using an Amicon Ultra centrifugal filter (MW cutoff, 100 kDa; Merck Millipore, Billerica, MA, USA). Pht without the PVP coating (Pht/PVP weight ratio of 1:0) was prepared using the method described above. The developed Pht@PVP MC was denoted by the MW of PVP (e.g., Pht@PVP10k MC for Pht encapsulated in 10 kDa PVP). To determine the loading efficiency (LE) of Pht@PVP MC, unloaded Pht was separated using an Amicon Ultra centrifugal filter and analyzed using GC-ToFMS with the aforementioned settings. Equation (1) was used to calculate the LE:

(1) $$\mathrm{LE}\;\left(\%\right)=\left(\frac{\mathrm{initial}\;\mathrm{amount}\;\mathrm{of}\;\mathrm{Pht}\;-\;\mathrm{unloaded}\;\mathrm{amount}\;\mathrm{of}\;\mathrm{Pht}}{\mathrm{initial}\;\mathrm{amount}\;\mathrm{of}\;\mathrm{Pht}}\right)\times 100$$

Hydrodynamic diameter, polydispersity index (PDI), and zeta potential of the Pht@PVP MC were measured using a Zetasizer (ELSZ-2000; Otsuka Electronics Co., Ltd., Osaka, Japan). The morphology of the MC was visualized using TEM with a JEM-2100Plus HR instrument (JEOL, Tokyo, Japan) at an acceleration voltage of 200 kV. For TEM analysis, 10 μL of 1 mg/mL MC solution was dropped twice onto a copper grid and allowed to dry at 25 °C for 3 d.

### 2.3. Stability Analysis of Pht@PVP MC

The stability of Pht@PVP360k MC and Pht@PVP1300k MC was analyzed for 2 weeks in a biological buffer (PBS) at 37 °C and 100 rpm. The hydrodynamic diameters and PDIs were analyzed weekly using a Zetasizer.

### 2.4. Antibacterial Activity of Pht@PVP MC

A previously described colony-counting method was used to quantitatively evaluate the antibacterial activity of Pht@PVP MC against *S. aureus* ATCC 6538 [23]. Luria–Bertani (LB) agar plates containing 1.5% agar (BD Difco, Sparks, MD, USA) were prepared for bacteria culture at 37 °C, and a single colony of *S. aureus* was inoculated in LB broth and cultured for 12 h at 37 °C before use. The culture was diluted with fresh LB broth until the optical density (OD_600_) reached 0.1. Pht, Pht@PVP360k MC, and Pht@PVP1300k MC (0.1% *v*/*v*) were subsequently added to each culture and incubated for 24 h to evaluate the bacterial growth inhibition. DI water was added to the negative control group. The suspensions were diluted to a factor of 10^8^ after incubation, and 100 μL of each test suspension was spread on an agar plate using glass beads. The colonies that appeared after 12 h of incubation were counted to determine *S. aureus* viability.

### 2.5. Cell Culture

NIH 3T3 mouse normal fibroblast cells and RAW264.7 macrophage cells were purchased from the Korean Cell Line Bank (Seoul, Republic of Korea) for in vitro assays. The cells were cultured at 37 °C under 5% CO_2_ in 10% fetal bovine serum (FBS; Gibco, Grand Island, NY, USA) and 1% antibiotic–antimycotic solution (AA; Thermo Fisher Scientific, Waltham, MA, USA), supplemented with Dulbecco’s modified Eagle’s medium (DMEM, Gibco).

### 2.6. In Vitro Cytotoxicity of Pht@PVP MC

The in vitro cytotoxicity of Pht@PVP MC was tested in NIH 3T3 fibroblasts. The cells were seeded in a 96-well plate at a density of 10,000 cells/well and incubated for 12 h at 37 °C in a humidified atmosphere with 5% CO_2_. Pht@PVP360k MC (1:4) at different concentrations, 10 to 500 μg/mL, was added to the cells, which were then incubated for 24 h. Cell Counting Kit-8 (CCK8; Dojindo Laboratories, Kumamoto, Japan) was used to analyze cell viability as described previously [24]. A microplate reader (VICTOR X5; PerkinElmer, Waltham, MA, USA) was used to measure absorbance at 450 nm. The cell viability of the control group was considered 100%, whereas that of Pht@PVP MC was evaluated as a relative percentage of the control.

### 2.7. In Vitro Antioxidant and Anti-Inflammatory Activities of Pht@PVP MC

The in vitro antioxidant activity of Pht@PVP360k MC (1:4) was evaluated in H_2_O_2_-stimulated NIH 3T3 cells using DMEM containing 10% FBS as the cell culture medium. Cells were seeded in a 96-well plate at a density of 10,000 cells/well and incubated for 12 h at 37 °C. Thereafter, Pht@PVP360k MC (1:4) at 10 and 100 μg/mL was added to the cells stressed with H_2_O_2_ (10 μM). The negative control groups were treated with cell culture medium only, representing the inherent cellular ROS levels. The increase in ROS levels upon H_2_O_2_ treatment was analyzed using H_2_DCFDA, which oxidizes into fluorescent 2′,7′-dichlorofluorescein in the presence of ROS. A microplate reader was used to detect fluorescence intensity at excitation and emission wavelengths of 485 and 535 nm, respectively. 

The inhibition of nitric oxide (NO) production in LPS-activated RAW264.7 macrophage cells after treatment with Pht@PVP360k MC (1:4) was tested to determine its anti-inflammatory efficacy [25]. LPS (100 ng/mL) was used as the oxidative stress agent. Pht@PVP360k MC (1:4) was used for NO inhibition at concentrations of 100 and 1000 μg/mL. After 24 h of incubation, the supernatant was collected and reacted with Griess reagent to detect NO. The absorbance was measured at 560 nm using a microplate reader, and the NO concentration was calculated relative to that of the control group.

Further, the in vitro antioxidant and anti-inflammatory activities of Pht@PVP360k MC (1:4) were analyzed after 2 weeks of storage in a biological buffer at 37 °C and 100 rpm. A comparison between its biological efficacies before and after storage was made to evaluate the stability of encapsulated Pht.

### 2.8. Statistical Analysis

All experiments were performed in triplicate (*n* = 3), and the resulting data are expressed as mean ± standard deviation. The significance of differences in data was evaluated using Student’s *t*-test and one-way *ANOVA*. Differences were considered statistically significant at *p* < 0.05.

## 3. Results and Discussion

### 3.1. Physicochemical Properties of Pht@PVP MC

**Figure 1:**
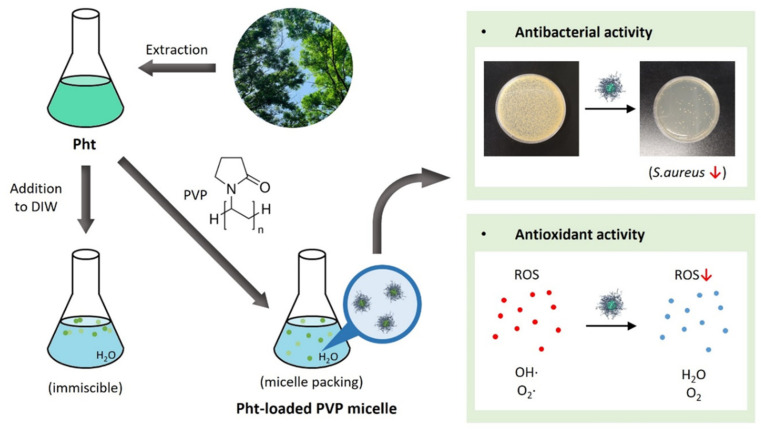
Schematic representation of phytoncide-loaded polyvinylpyrrolidone micelles. Abbreviations: Pht, phytoncide; PVP, polyvinylpyrrolidone; DIW, deionized water; ROS, reactive oxygen species.

**Figure 2:**
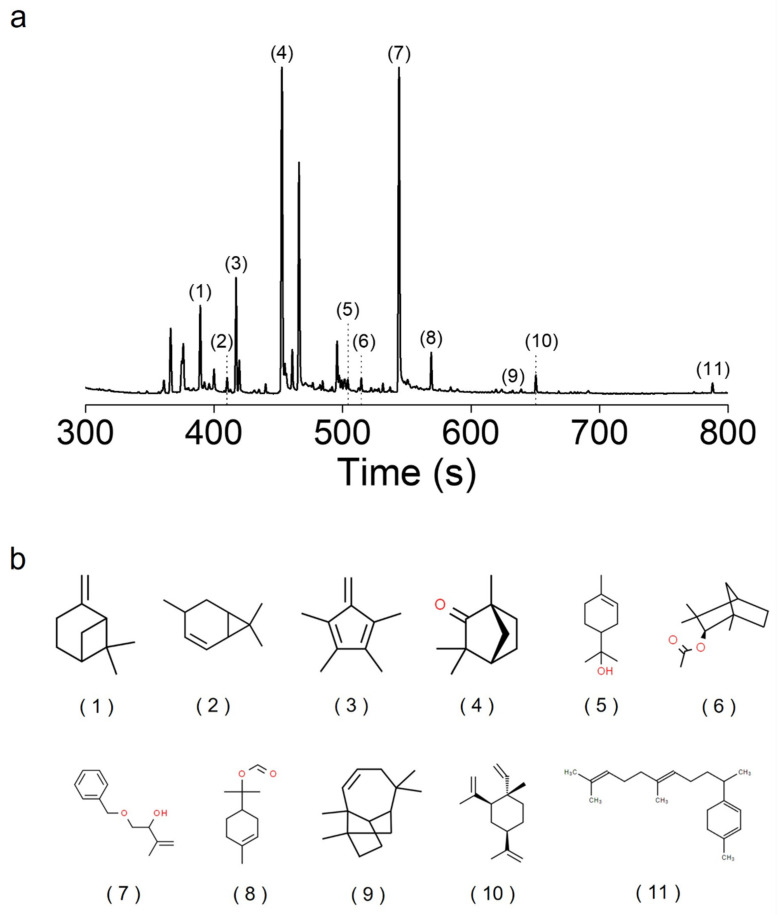
Analysis of phytoncide using gas chromatography coupled with time-of-flight mass spectrometry (GC-ToFMS). (**a**) GC-ToFMS chromatogram of phytoncide, including (1) L-β-pinene, (2) 4-carene, (3) 1,2,3,4 tetramethylfulvene, (4) L-fenchone, (5) α-terpineol, (6) β-fenchyl acetate, (7) 4-benzyloxy-3-hydroxy-2-methyl-1-butene, (8) terpinyl formate, (9) longifolene, (10) β-elemene, and (11) geranyl-α-terpinene, and (**b**) their chemical structures.

**Figure 3:**
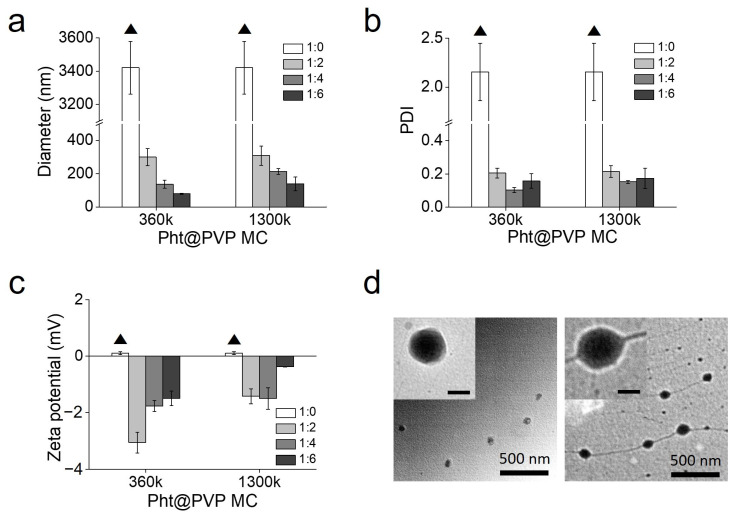
Characterization of phytoncide-encapsulated polyvinylpyrrolidone micelles (Pht@PVP MC) at different Pht/PVP weight ratios. (**a**) Hydrodynamic diameter, (**b**) polydispersity index (PDI), and (**c**) zeta potential of Pht@PVP MC. ▲ denotes the partial aggregation of Pht@PVP MC. (**d**) Transmission electron microscopy images of Pht@PVP360k MC (**left**) and Pht@PVP1300k MC (**right**). The Pht/PVP weight ratio used was 1:4. Inset scale bar: 50 nm. Abbreviations: Pht@PVP MC, phytoncide-encapsulated polyvinylpyrrolidone micelles; PDI, polydispersity index.

Many different templating materials have been used to encapsulate hydrophobic and volatile EOs. Rajkumar et al. used chitosan nanoparticles as a delivery system for peppermint EO [18]. The encapsulation of peppermint oil in the nanoparticles controlled the release rate and enhanced insecticidal activity against stored grain pests. In a study by Almeida et al., the physicochemical characteristics of *Cymbopogon citratus* EO were improved when loaded in poly(lactic-co-glycolic) acid nanoparticles [19]. This facilitated the controlled release of the EO and enhanced biocompatibility. Kapustová et al. reported a stronger antimicrobial efficacy of EOs after encapsulation in poly(ε-caprolactone) nanocapsules [20]. For use in beverages with better antioxidant and sensory properties, *Rosa damascena* EO has been encapsulated in β-cyclodextrin via host-guest complexation [26]. Furthermore, lipid-based nanocarriers, including lecithin and rhamnolipid, have been investigated for their ability to encapsulate hydrophobic plant by-products and vitamins [27,28]. In this study, the aqueous solubility of Pht increased upon the formation of noncovalent complexes with PVP, as depicted in Figure 1. Prior to encapsulation in PVP, Pht was characterized using GC-ToFMS, which revealed the presence of L-β-pinene, 4-carene, L-fenchone, α-terpineol, β-fenchyl acetate, terpinyl formate, longifolene, β-elemene, and geranyl-α-terpinene. Figure 2 illustrates the chromatogram of Pht and the chemical structures of the identified compounds, and Table S1 lists the compounds identified using GC-ToFMS with area % values. PVP contains hydrophilic polar amide moieties in its pyrrolidone ring and hydrophobic nonpolar methylene and methine groups within the ring and along its backbone [29]. This unique feature facilitates interactions with low-MW substances through hydrogen bonding and polar or hydrophobic attraction [30]. Notably, PVP has demonstrated its effectiveness as a carrier for poorly water-soluble curcumin from natural sources, enhancing its release rate and bioavailability [31]. Sui et al. developed fucoxanthin-based nanomedicines for targeted cancer therapy via nanoencapsulation in PVP nanoparticles [32]. PVP is considered a promising templating agent in food and pharmaceutical engineering [33]. Ozkan et al. co-precipitated poorly water-soluble flavonoids, such as quercetin and rutin, with PVP. PVP also facilitates pharmaceutical and nutraceutical applications of flavonoid compounds with antioxidant and nutritional benefits as supplements and functional foods. The adhesion between Pht and PVP is contingent on the MW of PVP; hence, an optimal MW for the micellar solubilization of Pht should be explored. Four types of PVP were tested for Pht encapsulation, as indicated in Table 1. Oil droplets were observed when insoluble Pht was added to DI water, which were marked as “Fail”. Low-molecular-weight PVP (MW = 10 and 40 kDa) did not adequately stabilize Pht, whereas high-molecular-weight PVP (360 and 1300 kDa) effectively encapsulated the hydrophobic Pht. However, a high PVP concentration (Pht:PVP weight ratio 1:6) resulted in a highly viscous Pht@PVP MC solution, hindering its practical use for biomedical applications. Pinene was scarcely detected in unloaded Pht compared with that in encapsulated Pht (Figure S1). The LE of Pht@PVP MC was > 99%, indicating the potential of PVP MC as a natural conservator. The hydrodynamic diameters of Pht@PVP360k MC and Pht@PVP1300k MC were small at a high PVP concentration, whereas that of bare Pht was 3421 ± 159 nm, forming partial aggregates (Figure 3a). The size of Pht@PVP360k MC decreased from 301 ± 51 to 80 ± 3 nm with increasing PVP content, whereas that of Pht@PVP1300k MC ranged from 310 ± 58 to 139 ± 42 nm. The PDIs of Pht@PVP MC was <0.3, indicating homogeneous size (Figure 3b). The zeta potentials of Pht@PVP MC represent their surface charges and were nearly neutral in the presence of neutral PVP polymer (Figure 3c). The spherical morphology of Pht@PVP360k MC (1:4) and Pht@PVP1300k MC (1:4) was observed using TEM (Figure 3d), with the MC size slightly smaller than that measured using the Zetasizer. This size discrepancy may be attributed to the shrinkage of PVP MC during TEM sampling on the copper grid. 

### 3.2. Stability of Pht@PVP MC

**Figure 4:**
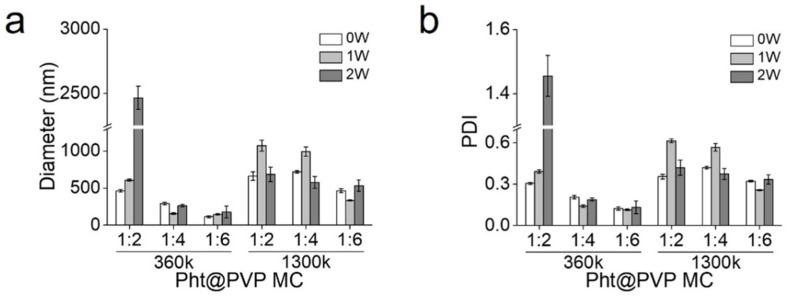
Stability analysis of phytoncide-encapsulated polyvinylpyrrolidone micelles (Pht@PVP MC). (**a**) Hydrodynamic diameter and (**b**) polydispersity index (PDI) of Pht@PVP360k MCs and Pht@PVP1300k MCs after 2 weeks of storage in biological buffer at 37 °C and 100 rpm. Abbreviations: Pht@PVP MC, phytoncide-encapsulated polyvinylpyrrolidone micelles; PDI, polydispersity index.

The hydrodynamic diameter and PDI of Pht@PVP360k MC and Pht@PVP1300k MC were monitored for 2 weeks in a biological buffer to analyze their stability under physiological conditions. PVP serves as a prominent stabilizer, protecting the nanoparticles from aggregation through steric hindrance and electrostatic interactions between its hydrophobic carbon chains and hydrophilic amide moieties [34,35]. Relying on the stabilizing effect of PVP, the size of Pht@PVP MC was maintained for 2 weeks without significant changes (Figure 4a). However, Pht@PVP360k MC (1:2) exhibited considerable enlargement, potentially due to the destabilization of its micellar system. As discussed in a previous report, the weight ratio of the encapsulating polymer strongly influences MC stability, depending on the differences in solubility and the degree of Pht dispersion in the PVP MCs [36]. Similar trends were observed for the PDIs, which remained stable at appropriate chain lengths and concentrations of PVP (Figure 4b).

### 3.3. Antibacterial Activity of Pht@PVP MC

**Figure 5:**
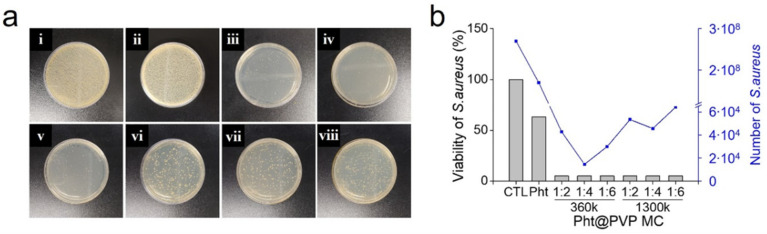
Antibacterial activity of Pht@PVP MC. (**a**) Photographs of *S. aureus* (**i**) without treatment (CTL, control), and treatment with (**ii**) Pht, (**iii**–**v**) Pht@PVP360k MC, and (**vi**–**viii**) Pht@PVP1300k MC at Pht/PVP weight ratios of 1:2, 1:4, and 1:6, respectively; (**b**) viability and colony count of *S. aureus* after treatment with Pht@PVP MC. Abbreviations: CTL, control; Pht, phytoncide; Pht@PVP MC, phytoncide-encapsulated polyvinylpyrrolidone micelles.

The antibacterial activity of Pht@PVP MC was assessed against the gram-positive bacterium *S. aureus*. Pht is known for its excellent antibacterial activity [37]. EOs act as effective antibacterial agents by disrupting phospholipids in cell membranes and enhancing cell wall permeability by interacting with enzymes [38]. Notably, promising antibacterial efficacy of *C. obtusa* EO against foodborne pathogens has demonstrated previously [39]. Terpineol considerably inhibits the growth of various pathogenic bacteria both in vitro and in vivo. Therefore, Pht exerted an inhibitory effect on *S. aureus* growth in a concentration-dependent manner (Figure S2). This finding is consistent with the results of Shin et al., who revealed the strong antimicrobial activity of Pht embedded in PVA nanofibers against both *S. aureus* and *E. coli* [40]. As shown in Figure 5, 0.1% *v*/*v* Pht reduced the viability of *S. aureus* to 63%. At the same concentration, Pht@PVP MC exhibited significantly higher antibacterial activity. After treatment with Pht@PVP MC, the viability of *S. aureus* was below 99%, indicating a substantial enhancement in Pht bioavailability through micellar solubilization. The number of *S. aureus* colonies in the negative control group was approximately 2.7 × 10^8^, decreasing to 1.46 × 10^4^ and 6.42 × 10^4^ in the presence of Pht@PVP MC. Although the antibacterial properties of inactive PVP have not been previously reported, the enhanced bioavailability of Pht via micelle packing resulted in the synergistic antibacterial activity of Pht@PVP MC. An increase in the MW and concentration of PVP decreased the antibacterial activity; fewer colonies were observed on agar plates treated with Pht@PVP360k MC than on those treated with Pht@PVP1300k MC. This result could be attributed to the weakening of the interaction with and decrease in the bioavailability of Pht to bacteria upon strengthening the PVP-shielding effect. Moreover, the antibacterial activity of Pht@PVP360k MC (1:2) was low, possibly because of its unstable micellar structure. In conclusion, Pht@PVP360k MC (1:4) enhanced the antimicrobial activity of Pht by enabling adequate solubilization and stabilization.

### 3.4. In Vitro Cytotoxicity, Antioxidant, and Anti-Inflammatory Activity of Pht@PVP MC

**Figure 6:**
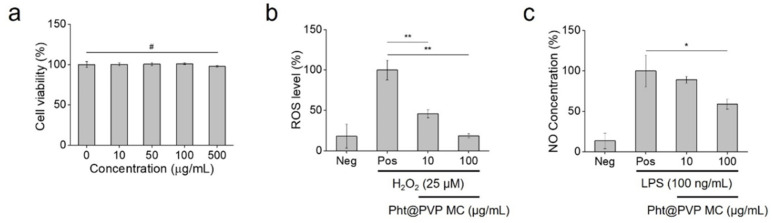
In vitro biocompatibility and efficacy of Pht@PVP360k MC (Pht/PVP ratio = 1:4). (**a**) Cytotoxicity of Pht@PVP360k MC in NIH 3T3 fibroblasts. (**b**) In vitro antioxidant activity and (**c**) in vitro anti-inflammatory efficacy of Pht@PVP MC at different concentrations. Values are presented as mean ± standard deviation; they were assessed using Student’s *t*-test and one-way ANOVA. # *p* > 0.05, * *p* < 0.05, and ** *p* < 0.01. Abbreviations: ROS, reactive oxygen species; NO, nitric oxide; Neg, negative control; Pos, positive control; LPS, lipopolysaccharide from *E. coli* O111:B4; Pht@PVP MC, phytoncide-encapsulated polyvinylpyrrolidone micelles.

The biocompatibility of Pht@PVP MC was evaluated in vitro by monitoring the viability of NIH 3T3 fibroblasts after MC treatment. The U.S. Food and Drug Administration has approved the biomedical applications of PVP [41], and Pht has been shown to be non-cytotoxic to normal fibroblast cells [42,43]. Consistently, Pht@PVP MC was non-toxic to NIH 3T3 cells, with cell viability exceeding 95% after treatment with 10–500 μg/mL Pht@PVP MC (Figure 6a). The reduction in cell viability caused by Pht@PVP MC was negligible (# *p* > 0.05), indicating its potential for biomedical applications without cytotoxic or adverse effects. The biocompatible Pht@PVP MC exhibited excellent antioxidant and anti-inflammatory activities. As shown in Figure 6b, 10 and 100 μg/mL Pht@PVP MC reduced ROS levels from 100% to 45.8% and 18.6%, respectively. Considering that the normal intracellular ROS level in the negative control group was 18.0%, it is noteworthy that Pht@PVP MC significantly inhibited ROS production in H_2_O_2_-stimulated cells (** *p* < 0.01). The in situ antioxidant activity of EOs from trees has been previously reported, and the major components of Pht, including pinene and carene, have been identified as promising antioxidant candidates for food and medical purposes [44,45,46]. Furthermore, Pht@PVP MC has been confirmed to be an effective inhibitor of NO, a major pro-inflammatory mediator. When Pht@PVP MC was added to LPS-stimulated RAW264.7 cells, NO production was reduced by 58.8% (Figure 6c). This observation aligns with the findings of Kang et al., who demonstrated the attenuating effect of Pht on LPS-induced inflammatory responses [47]. Pht suppresses the upregulation of NO synthase and cyclooxygenase-2 expression, thereby exerting beneficial effects on gastrointestinal inflammation [48,49]. It can also stimulate the immune system against inflammatory and infectious diseases. It has been reported that stress hormone levels decrease upon Pht exposure based on natural killer cell activity [8]. Further, Pht@PVP MC maintained its in vitro biological efficacies even after 2 weeks of storage. As shown in Figure S3, it exhibited excellent antioxidant and anti-inflammatory activities with no degradation of Pht (# *p* > 0.05). These results suggest the application of Pht@PVP MC as a promising nutritional and therapeutic agent. Furthermore, dietary supplementation with antioxidant and anti-inflammatory Pht has been found to be beneficial for the meat quality of finishing pigs and egg production in laying hens [50,51]. It has been demonstrated that the antioxidative potential of Pht reduced the oxidative stress response and improved meat quality. Pht reportedly exhibited immunomodulatory effects in LPS-injected hens, facilitating the potential use of Pht@PVP MC as a functional feed additive. However, it requires further improvement to produce in a large scale for its industrial usage.

## 4. Conclusions

Plant-derived Pht was encapsulated in PVP micelles to enhance the bioavailability and efficacy of natural antioxidants and biocides for nutraceutical applications. An increase in the amount of high-molecular-weight PVP successfully dispersed Pht without forming insoluble aggregates. Pht@PVP MC exhibited uniform nanometer size distribution, neutral surface charge, and spherical morphology. Stable for 2 weeks under physiological conditions, Pht@PVP MC maintained its size and PDI owing to the stabilizing effect of PVP. In comparison to insoluble Pht, Pht@PVP MC exhibited strong antibacterial activity against *S. aureus*, indicating enhanced bioavailability for bacterial growth inhibition. The degree of efficacy enhancement correlated with the concentration and MW of PVP. Additionally, the in vitro analysis revealed that Pht@PVP MC reduced oxidative stress and inflammation without inducing cytotoxic effects. These findings suggest that Pht@PVP MC significantly improved the stability and efficiency of Pht. Consequently, Pht@PVP MC is considered a safe and effective candidate for antibacterial, antioxidant, and anti-inflammatory nanomedicines.

## Figures and Tables

**Table 1:** Solubilization of phytoncide in various weight ratios of polyvinylpyrrolidone of different molecular weights.

Pht:PVP		Molecular Weight (kDa) of PVP			
		10	40	360	1300
Solubilization of Pht	1:0	Fail	Fail	Fail	Fail
	1:2	Fail	Fail	Pass	Pass
	1:4	Fail	Fail	Pass	Pass
	1:6	Fail	Fail	Pass (highly viscous)	Pass (highly viscous)

Abbreviations: kDa, kilodalton; Pht, phytoncide; PVP, polyvinylpyrrolidone.

## Data Availability

Data will be made available on request.

## Supplementary Materials

The following supporting information can be downloaded at: https://www.mdpi.com/article/10.3390/antiox13091056/s1 Table S1. Phytoncide compounds were identified using gas chromatography device coupled with a time-of-flight mass spectrometry detector (GC-ToFMS); Figure S1. Analysis of the loading efficiency of phytoncide in polyvinylpyrrolidone micelles using gas chromatography device coupled with time-of-flight mass spectrometry detector (GC-ToFMS). GC-ToFMS chromatogram of L-β-pinene in phytoncide was used to calculate the loading efficiency; Figure S2. Antibacterial activity of phytoncide (Pht) against *Staphylococcus aureus* at different concentrations; Figure S3. (a) In vitro antioxidant and (b) anti-inflammatory activities of Pht@PVP360k MC (Pht/PVP ratio = 1:4) before and after 2 weeks of storage in a biological buffer. ROS, reactive oxygen species; NO, nitric oxide; Neg, negative control; Pos, positive control; LPS, lipopolysaccharides from *E. coli* O111:B4. Values are presented as the mean ± SD and were assessed using Student’s *t*-test. # *p* > 0.05.
