# Supplementary material for: Phytochemical-Based Nanoantioxidants Stabilized with Polyvinylpyrrolidone for Enhanced Antibacterial, Antioxidant, and Anti-Inflammatory Activities
Source: Antioxidants (Basel). 2024 Aug 30;13(9):1056. doi: 10.3390/antiox13091056 (PMC11429192; doi:10.3390/antiox13091056)
Supplement: Supplementary file 1 [file antioxidants-13-01056-s001.zip › antioxidants-3144867-supplementary.pdf]

Supporting Information

Phytochemical-Based Nanoantioxidants Stabilized with Polyvinylpyrrolidone for Enhanced Antibacterial, Antioxidant, and Anti-Inflammatory Activities

Hyeryeon Oh [1,2], Jin Sil Lee [1,2], Hyojung Park [1], Panmo Son [1,3], Byoung Seung Jeon [1], Sang Soo Lee [4], Daekyung Sung [1], Jong-Min Lim [4,5,*] and Won Il Choi [1,*]

[1] Bio-Convergence Materials R&D Division, Korea Institute of Ceramic Engineering and Technology, 202 Osongsaengmyeong 1-ro, Heungdeok-gu, Cheongju 28160, Republic of Korea
[2] School of Materials Science and Engineering, Gwangju Institute of Science and Technology, 123 Cheomdangwagi-ro, Buk-gu, Gwangju 61005, Republic of Korea
[3] Department of Applied Bioengineering, Graduate School of Convergence Science and Technology, Seoul National University, 1 Gwanak-ro, Gwanak-gu, Seoul 08826, Republic of Korea
[4] Department of Electronic Materials, Devices, and Equipment Engineering, Soonchunhyang University, 22 Soonchunhyang-ro, Shinchang-myeon, Asan 31538, Republic of Korea
[5] Department of Chemical Engineering, Soonchunhyang University, 22 Soonchunhyang-ro, Shinchang-myeon, Asan 31538, Republic of Korea
* Correspondence: jmlim@sch.ac.kr (J.-M.L.); choi830509@kicet.re.kr (W.I.C.); Tel.: +82-41-530-4961 (J.-M.L.); +82-43-913-1513 (W.I.C.)

Table S1. Phytoncide compounds were identified using a gas chromatography device coupled with a time-of-flight mass spectrometry detector (GC-ToFMS).

No.	Retention time (s)	Name	Area %
(1)	389	L-β-pinene	45,918,810,200
(2)	410	4-Carene, (1S, 3R, 6R)-(–)-	5,856,308,200
(3)	417	1,2,3,4 tetramethylfulvene	85,437,431,300
(4)	453	L-Fenchone	224,975,252,300
(5)	501	α-Terpineol	1,571,234,000
(6)	515	β-Fenchyl acetate	4,013,598,300
(7)	544	4-Benzyloxy-3-hydroxy-2-methyl-1-butene	130,762,949,500
(8)	569	Terpinyl formate	8,337,096,800
(9)	632	Longifolene-(V4)	770,058,500
(10)	650	β-elemene	2,392,590,000
(11)	788	Geranyl-α-Terpinene	1,177,063,500

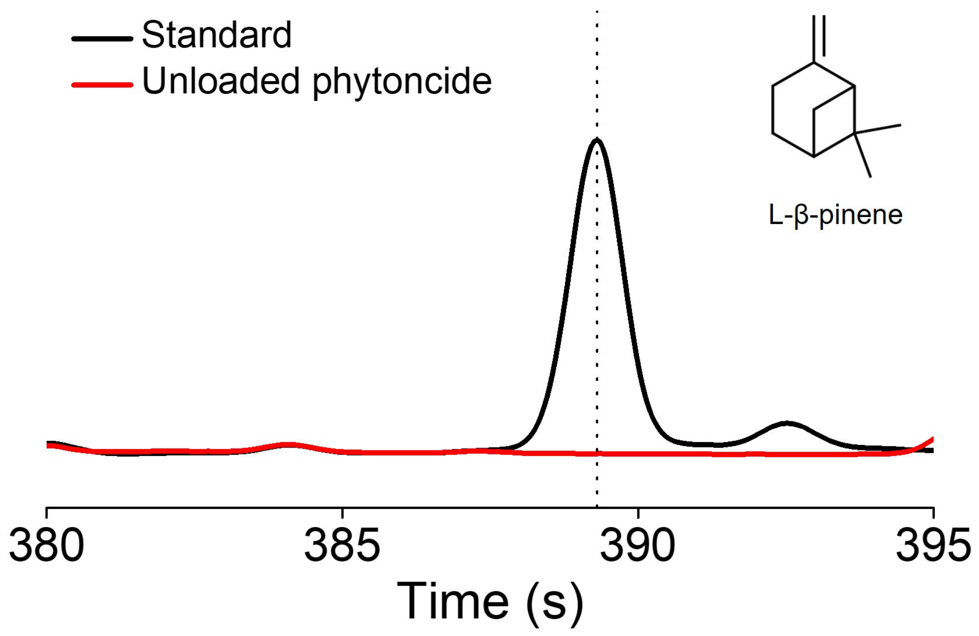

Figure S1. Analysis of the loading efficiency of phytoncide in polyvinylpyrrolidone micelles using gas chromatography device coupled with time-of-flight mass spectrometry detector (GC-ToFMS). GC-ToFMS chromatogram of L-β-pinene in phytoncide was used to calculate the loading efficiency.

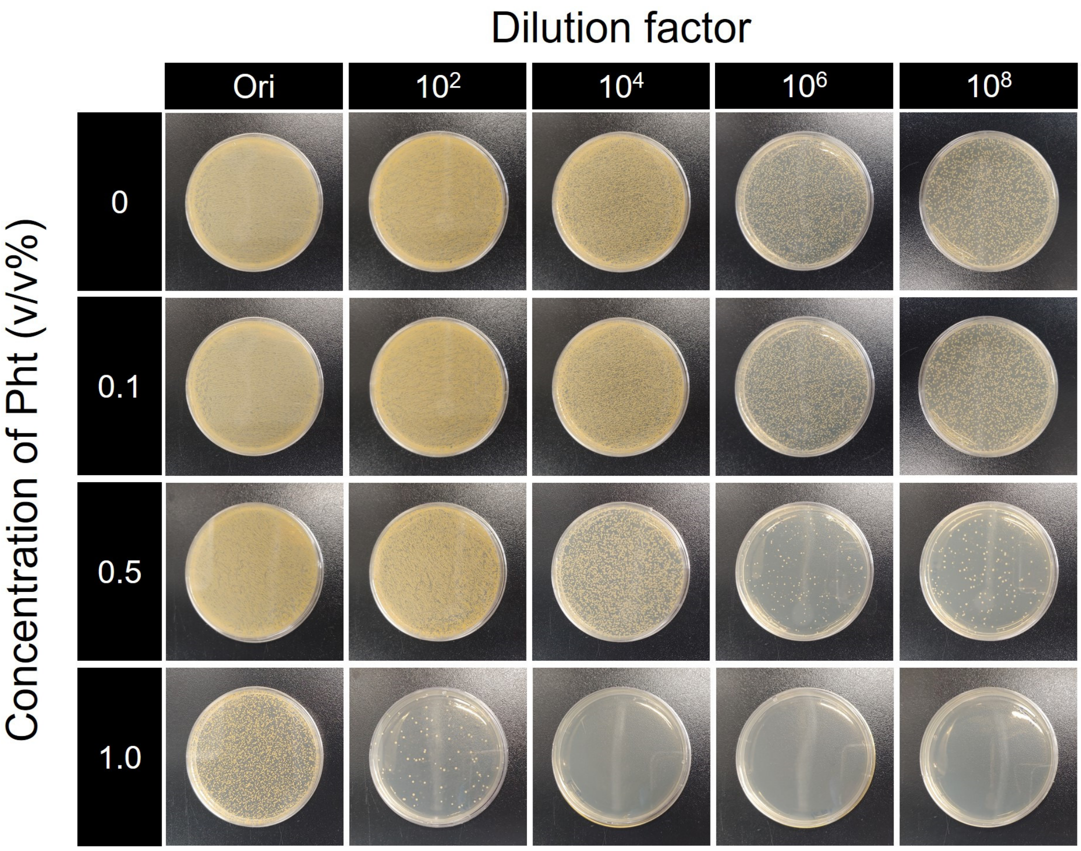

Figure S2. Antibacterial activity of phytoncide (Pht) against *Staphylococcus aureus* at different concentrations. Photographs of *S. aureus* colonies diluted by different factors.

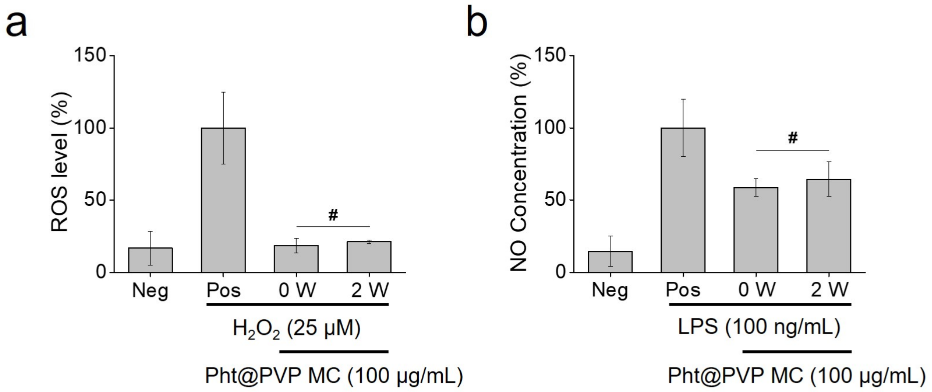

Figure S3. (a) *In vitro* antioxidant and (b) anti-inflammatory activities of Pht@PVP360k MC (Pht/PVP ratio = 1:4) before and after two weeks of storage in a biological buffer. ROS, reactive oxygen species; NO, nitric oxide; Neg, negative control; Pos, positive control; LPS, lipopolysaccharides from *E. coli* O111:B4. Values are presented as the mean ± SD and were assessed using Student's *t*-test. #$p > 0.05$.
